# Comparison of carbon ion radiotherapy and transarterial chemoembolization for unresectable solitary hepatocellular carcinoma >3 cm: a propensity score–matched analysis

**DOI:** 10.1093/jrr/rraf026

**Published:** 2025-05-13

**Authors:** Taito Fukushima, Satoshi Kobayashi, Hiroyuki Katoh, Tomomi Hamaguchi, Yuichiro Tozuka, Yasutsugu Asai, Shun Tezuka, Makoto Ueno, Manabu Morimoto, Junji Furuse, Shin Maeda

**Affiliations:** Department of Gastroenterology, Kanagawa Cancer Center, 2-3-2 Nakao, Asahi-ku, Yokohama, Kanagawa 241-8515, Japan; Department of Gastroenterology, Kanagawa Cancer Center, 2-3-2 Nakao, Asahi-ku, Yokohama, Kanagawa 241-8515, Japan; Division of Radiation Oncology, Kanagawa Cancer Center, 2-3-2 Nakao, Asahi-ku, Yokohama, Kanagawa 241-8515, Japan; Department of Gastroenterology, Kanagawa Cancer Center, 2-3-2 Nakao, Asahi-ku, Yokohama, Kanagawa 241-8515, Japan; Department of Gastroenterology, Kanagawa Cancer Center, 2-3-2 Nakao, Asahi-ku, Yokohama, Kanagawa 241-8515, Japan; Department of Gastroenterology, Kanagawa Cancer Center, 2-3-2 Nakao, Asahi-ku, Yokohama, Kanagawa 241-8515, Japan; Department of Gastroenterology, Kanagawa Cancer Center, 2-3-2 Nakao, Asahi-ku, Yokohama, Kanagawa 241-8515, Japan; Department of Gastroenterology, Kanagawa Cancer Center, 2-3-2 Nakao, Asahi-ku, Yokohama, Kanagawa 241-8515, Japan; Department of Gastroenterology, Kanagawa Cancer Center, 2-3-2 Nakao, Asahi-ku, Yokohama, Kanagawa 241-8515, Japan; Gastroenterological Center, Yokohama City University Medical Center, 4-57 Urafune-cho, Minami-ku, Yokohama, Kanagawa 232-0024, Japan; Department of Gastroenterology, Kanagawa Cancer Center, 2-3-2 Nakao, Asahi-ku, Yokohama, Kanagawa 241-8515, Japan; Department of Gastroenterology, Yokohama City University Graduate School of Medicine, 3-9 Fukuura, Kanazawa-ku, Yokohama, Kanagawa 236-0004, Japan

**Keywords:** carbon ion radiotherapy, hepatocellular carcinoma, propensity score, sequential treatment, systemic therapy, transarterial chemoembolization

## Abstract

This study aimed to compare outcomes between carbon ion radiotherapy (C-ion RT) and transarterial chemoembolization (TACE) in patients with unresectable solitary hepatocellular carcinoma (HCC) >3 cm. Fifty-eight patients who had been treated with C-ion RT (C-ion RT group) and 34 treated with TACE (TACE group) were retrospectively enrolled between January 2016 and December 2021. Propensity score matching was conducted to account for differences between the two groups. The median follow-up duration was 42.1 months for all patients. Propensity score matching successfully balanced the two groups with 29 patients matched to each group. The 3-year overall survival (OS), progression-free survival (PFS) and local control (LC) rates in the C-ion RT vs TACE groups were 75.9% vs 45.4%, 44.8% vs 16.1% and 85.2% vs 23.2%, respectively. The C-ion RT group showed better OS (hazard ratio [HR], 0.578 [95% confidence interval (CI): 0.295–1.132]; *P* = 0.106), PFS (HR, 0.460 [95% CI: 0.254–0.835]; *P* = 0.009) and LC (HR, 0.155 [95% CI: 0.062–0.390]; *P* < 0.001) than the TACE group. Multivariate analysis indicated that C-ion RT was significantly associated with increased PFS (HR, 0.562 [95% CI: 0.341–0.926]; *P* = 0.024) and LC (HR, 0.282 [95% CI: 0.150–0.528]; *P* < 0.001). C-ion RT provided better OS, PFS and LC than TACE in patients with solitary HCC >3 cm. This study indicated that C-ion RT is a possible alternative to TACE, which is the standard of care for patients with medium-to-large-sized HCCs.

## INTRODUCTION

Advances in therapeutic approaches have improved treatment outcomes for hepatocellular carcinoma (HCC). Nevertheless, HCC is the third leading cause of cancer-related mortality worldwide [1]. Although surgery is often the preferred choice for solitary HCC, radiofrequency ablation (RFA) is widely used as an alternative curative therapy in patients who refuse surgery or who are medically inoperable. However, RFA may be ruled out, and transarterial chemoembolization (TACE) can be chosen for several reasons, such as large tumor size and inaccessible location [2, 3]. Although TACE is recommended in several guidelines as a first-line treatment for patients with Barcelona Clinic Liver Cancer stage B HCC, its treatment efficacy is suboptimal for solitary HCC, which should be treated with curative intent [4–6].

In recent years, particle therapies, including proton beam radiotherapy and carbon ion radiotherapy (C-ion RT), have gained attention as alternatives to RFA and TACE. C-Ion RT has shown superior dose distribution characteristics attributed to the distal tail-off of the Bragg peak and the sharp lateral penumbra. These features enable the mitigation of damage to healthy liver tissues [7]. As a result, high-dose irradiation of the tumor is possible, enabling excellent local control (LC). Previous studies have reported that C-ion RT shows a notable LC rate and fewer complications in patients with HCC [8–14]. Therefore, C-ion RT may be a new and beneficial locoregional treatment alternative to TACE and other locoregional treatments.

To our knowledge, no study has compared C-ion RT and TACE in terms of outcomes for medium-to-large-sized HCCs. We focused on solitary HCC >3 cm because local recurrence rates for RFA increase with increasing tumor size [15, 16]. Moreover, the Barcelona Clinic Liver Cancer staging system and Japanese clinical practice guidelines for HCC (5th JSH-HCC guidelines) [4, 17] recommend RFA for patients with the largest HCC diameter being ≤3 cm and having ≤3 nodules.

Thus, locoregional treatment options other than TACE for medium-to-large-sized HCCs are limited. Tumor size is a prognostic factor for survival after TACE. As tumor size increases, the presence of extrahepatic collaterals limits treatment efficacy [18, 19]. Even for solitary HCC, larger tumor diameters are associated with poor tumor response and survival [20]. Therefore, alternative treatments to TACE are needed for HCC where curative treatment is challenging. The aim of this study was to compare the clinical outcomes of C-ion RT with those of TACE for solitary HCC >3 cm.

## MATERIALS AND METHODS

### Patients

This single-center retrospective study included patients with HCC who had been treated with C-ion RT or TACE between January 2016 and December 2021. Clinical information and follow-up data were obtained from medical records. The data cutoff date was 30 November 2024. The inclusion criteria were a diagnosis of HCC with pathological confirmation or typical HCC findings on computed tomography or magnetic resonance imaging; no prior C-ion RT or TACE; inoperability due to refusing surgery, their general health (age and comorbidities) or inadequate liver condition (liver function or tumor localization); and solitary HCC with a maximum tumor diameter >3 cm. The exclusion criteria were macrovascular invasion and extrahepatic metastasis, combination therapy with other treatments and additional coexisting malignancies. The medical records of 176 consecutive patients with HCC who had been treated with C-ion RT, and 217 patients who had been treated with TACE for the first time, were retrospectively reviewed. Based on the inclusion and exclusion criteria, 58 and 34 patients who had undergone C-ion RT and TACE, respectively, were included.

The work described has been carried out in accordance with The Code of Ethics of the World Medical Association (Declaration of Helsinki) for experiments involving humans, and all procedures were performed in compliance with relevant laws and institutional guidelines. The study has been approved by the Institutional Review Board of Kanagawa Cancer Center (approval date: 10 July 2023; reference number: 2023-57). The requirement for written informed consent was waived owing to the retrospective nature of the study. Patients were given the opportunity to opt out of the study.

### Carbon ion radiotherapy

Metallic fiducial markers were implanted alongside the target tumor as landmarks in all patients. Simulation and planning were based on four-dimensional computed tomography. Contrast-enhanced computed tomography (2-mm slice thickness) was used for treatment planning to ensure an optimal target definition. Gross tumor volume was defined as the area of solid macroscopic tumor contrast enhancement on computed tomography. Clinical target volume was defined as the gross tumor volume plus a margin of 5–10 mm, with modifications to exclude the gastrointestinal tract and portal vein. The planning target volume included the clinical target volume plus a margin of 5 mm, which accounted for organ motion and setup inaccuracies. The planning target volume (PTV) was also modified according to the organ at risk. The treatment plan aimed to cover 100% of the PTV with 95% of the prescribed dose. C-Ion RT treatment plans for all patients were created using Monaco for carbon-ion scanning (version 5.20; Elekta AB, Stockholm, Sweden). C-Ion RT treatment plans for all patients were performed using the spot scanning method. C-Ion RT was performed with respiration-gated irradiation, with the gate level set such that the target travel range was <5 mm from the four-dimensional computed tomography. The relative biological effectiveness-weighted absorbed dose to the PTV was 60 Gy in 4 or 12 fractions, considering the tumor size and location.

### Transarterial chemoembolization

After local anesthesia, the femoral artery was catheterized using the Seldinger technique by introducing a 4-French catheter (Selecon; Terumo, Tokyo, Japan) into the hepatic artery. Following this, a 1.9-French microcatheter (Progreat Σ; Terumo) was inserted into the feeder arteries of each tumor. After microcatheter placement, epirubicin hydrochloride and embolic agents were injected into the feeder arteries. From 2016 to 2019, HepaSphere 50–100 μm (Merit Medical Systems, South Jordan, UT, USA) was used for embolization. From 2020 to 2021, Embosphere 100–300 μm (Merit Medical Systems) was used for embolization. The injection was administered to near stasis, as indicated by the disappearance of contrast within 2–5 heartbeats [21]. On-demand TACE was performed in all patients. None of the patients were scheduled to undergo TACE.

### Assessment and follow-up

Patients underwent abdominal computed tomography or magnetic resonance imaging at 1–3 months after C-ion RT or TACE, and every 3 months thereafter. Radiological assessments were performed according to modified Response Evaluation Criteria in Solid Tumors [22]. LC was defined as the absence of any indication of local recurrence. Local recurrence was defined as either progressive disease (modified Response Evaluation Criteria in Solid Tumors) or the appearance of new lesions within the treatment field (irradiation field after C-ion RT and the embolization zone after TACE). Local recurrence was independently evaluated by the attending physician and radiation oncologist. In case of disagreement, the final decision was made by the attending physician. Patients treated with TACE showing residual lesions during follow-up imaging underwent additional TACE with residual lesions not classified as local recurrence. The starting date for calculating LC was defined as either the initiation date of C-ion RT or the date of the first TACE. Progression-free survival (PFS) was defined as the time from the date of C-ion RT or the first TACE to disease progression or death. Overall survival (OS) was defined as the time from the date of C-ion RT or the first TACE until death. Albumin–bilirubin scores were evaluated at the initiation of C-ion RT or TACE and 3 and 6 months thereafter.

### Statistical analysis

Continuous variables were analyzed using the Mann–Whitney *U* test. Categorical variables were analyzed using Fisher’s exact test or the chi-square test. A *P-*value <0.05 was considered statistically significant. OS, PFS and LC were estimated using the Kaplan–Meier method. Hazard ratios (HRs) with 95% confidence intervals (CIs) were calculated using Cox regression. Propensity score matching was used to minimize potential bias between the groups. Propensity scores were estimated using age, sex, Child–Pugh class, tumor size and alpha-fetoprotein. All variables were weighted equally in the matching process. Multivariate analysis was performed using a Cox proportional hazards model to identify prognostic factors influencing OS, PFS and LC. Five covariates (age, sex, Child–Pugh class, tumor size and alpha-fetoprotein) were selected that were deemed clinically significant in relation to OS, PFS and LC. Statistical analyses were performed using SPSS for Windows (version 25; IBM Corp., Armonk, NY, USA).

## RESULTS

### Clinical characteristics

The median follow-up at the time of data cutoff was 42.1 months. During follow-up, 26 and 25 patients in the C-ion RT and TACE groups died, respectively. Clinical characteristics are summarized in Table 1. Among the entire cohort, there was a significant difference between the two groups in terms of etiology (*P* = 0.038) and alpha-fetoprotein (*P* = 0.012). Propensity score matching successfully balanced the two groups by matching 29 patients to each group. In the matched cohort, no differences were observed between the two groups.

**Table 1:** Baseline characteristics

	Before PSM			After PSM		
	C-ion RT (*n* = 58)	TACE (*n* = 34)	*P-*value	C-ion RT (*n* = 29)	TACE (*n* = 29)	*P-*value
Age (years)	78 (70–84)	79 (74–82)	0.662	80 (75–82)	78 (73–82)	0.657
Sex						
Male	48	27	0.690	24	23	0.487
Female	10	7		4	6	
Etiology						
Viral	18	18	**0.038**	12	13	0.791
Non-viral	40	16		17	16	
ECOG-PS						
0	46	29	0.475	21	26	0.094
1	12	5		8	3	
Size (mm)	49 (38–70)	54 (41–80)	0.517	51 (40–70)	49 (37–77)	0.994
30–60	37	19	0.691	18	18	0.901
60–90	14	9		8	7	
>90	7	6		3	4	
Child–Pugh class						
A	53	29	0.365	26	26	1.000
B	5	5		3	3	
ALBI grade						
1	32	19	0.947	14	18	0.291
2	26	15		15	11	
Albumin (g/dL)	4.0 (3.7–4.2)	3.9 (3.6–4.2)	0.564	3.9 (3.5–4.1)	4.0 (3.6–4.2)	0.463
Total bilirubin (mg/dL)	0.8 (0.6–1.0)	0.7 (0.6–1.0)	0.311	0.8 (0.6–1.0)	0.7 (0.5–1.0)	0.288
ALT (IU/L)	27.0 (17–33)	30.0 (21–44)	0.293	27 (19–31)	30 (19–45)	0.315
AFP (ng/mL)	4.3 (2.7–13.6)	12.0 (6.3–78.1)	**0.012**	4.9 (3.2–91.6)	12.0 (6.3–78.1)	0.178
Previous treatment						
Yes	5	4	0.624	2	4	0.389
No	53	30		27	25	
RBE-weighted dose						
60 Gy in 4 fractions	40			21		
60 Gy in 12 fractions	18			8		
Reason for non-resectability						
Patient refused surgery	15	9	0.792	5	7	0.414
Patient’s general health	26	13		15	10	
Inadequate liver condition	17	12		9	12	

Values are presented as *n* or median (IQR). Bold values indicate statistical significance (*P* < 0.05). AFP = alpha-fetoprotein, ALBI = albumin–bilirubin, ALT = alanine transaminase, BCLC = Barcelona Clinic Liver Cancer, C-ion RT = carbon ion radiotherapy, ECOG-PS = Eastern Cooperative Oncology Group performance status, IQR = interquartile range (25th–75th percentile), PSM = propensity score matching, RBE = relative biological effectiveness, TACE = transarterial chemoembolization.

### Carbon ion radiotherapy treatment parameters

The dose–volume histogram parameters of C-ion RT were as follows: mean liver dose, 12.67 Gy (median, 11.29 Gy [range, 4.50–29.85 Gy]); V_20Gy_, 22.69% (median, 19.81% [range, 7.57–56.73%]).

### Overall survival, progression-free survival and local control

**Fig. 1:**
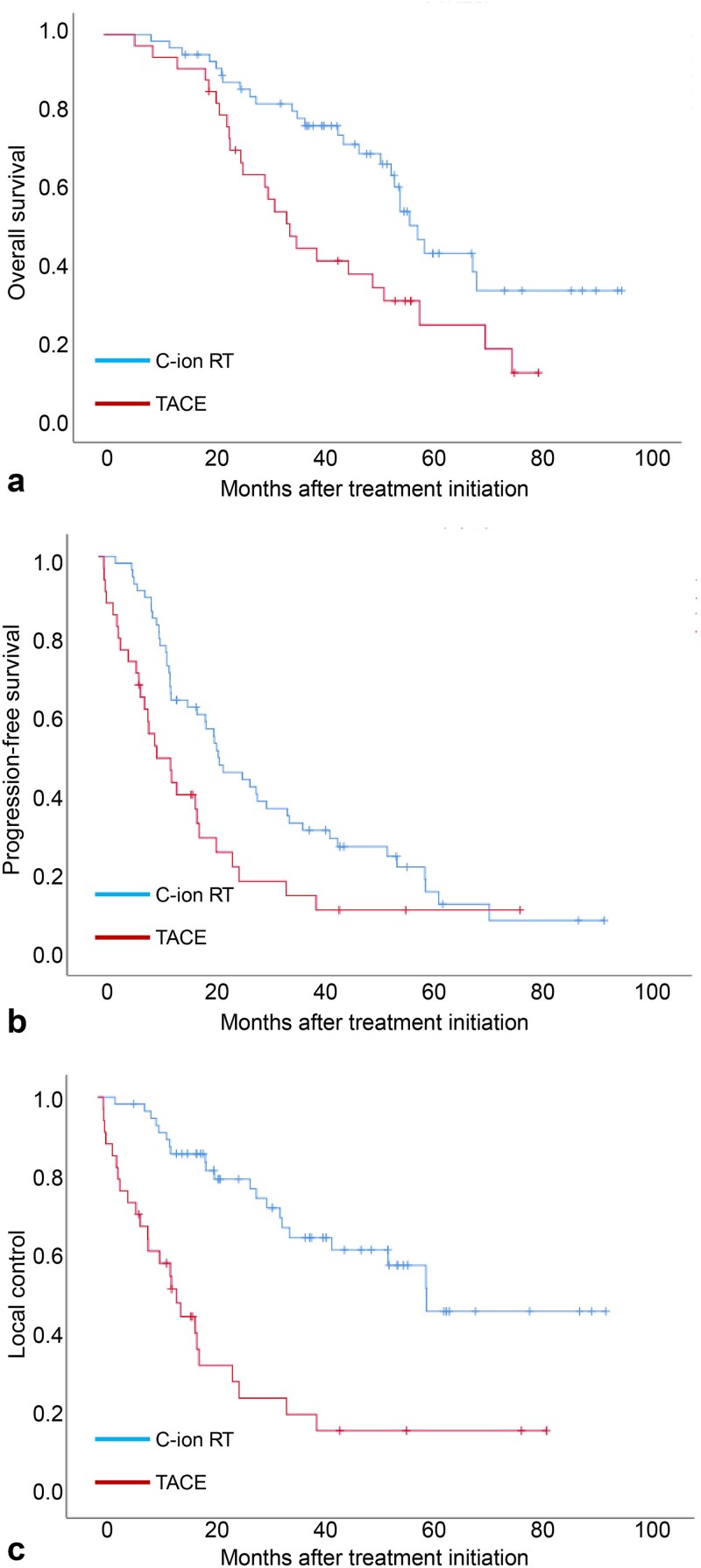
Kaplan–Meier estimates of (a) overall survival, (b) progression-free survival and (c) local control in the entire cohort. C-ion RT = carbon ion radiotherapy, TACE = transarterial chemoembolization.

The Kaplan–Meier curves for OS, PFS and LC in the entire cohort are shown in Fig. 1. The median OS in the C-ion RT group was longer than that in the TACE group (57.3 vs 34.0 months, respectively). The 1- and 3-year OS rates were 96.6% and 78.3% in the C-ion RT group, and 94.1% and 44.6% in the TACE group (HR, 0.470 [95% CI: 0.271–0.816]; *P* = 0.006), respectively. The median PFS in the C-ion RT group was longer than that in the TACE group (21.3 vs 10.3 months, respectively). The 1- and 3-year PFS rates were 75.4% and 32.8% in the C-ion RT group, and 49.2% and 14.5% in the TACE group (HR, 0.549 [95% CI: 0.342–0.881]; *P* = 0.012), respectively. The median time to LC in the C-ion RT group was longer than that in the TACE group (58.1 vs 13.9 months, respectively). The 1- and 3-year LC rates were 90.9% and 64.7% in the C-ion RT group, and 58.3% and 20.4% in the TACE group (HR, 0.260 [95% CI: 0.144–0.469]; *P* < 0.001), respectively.

### Overall survival, progression-free survival and local control after propensity score matching

**Fig. 2:**
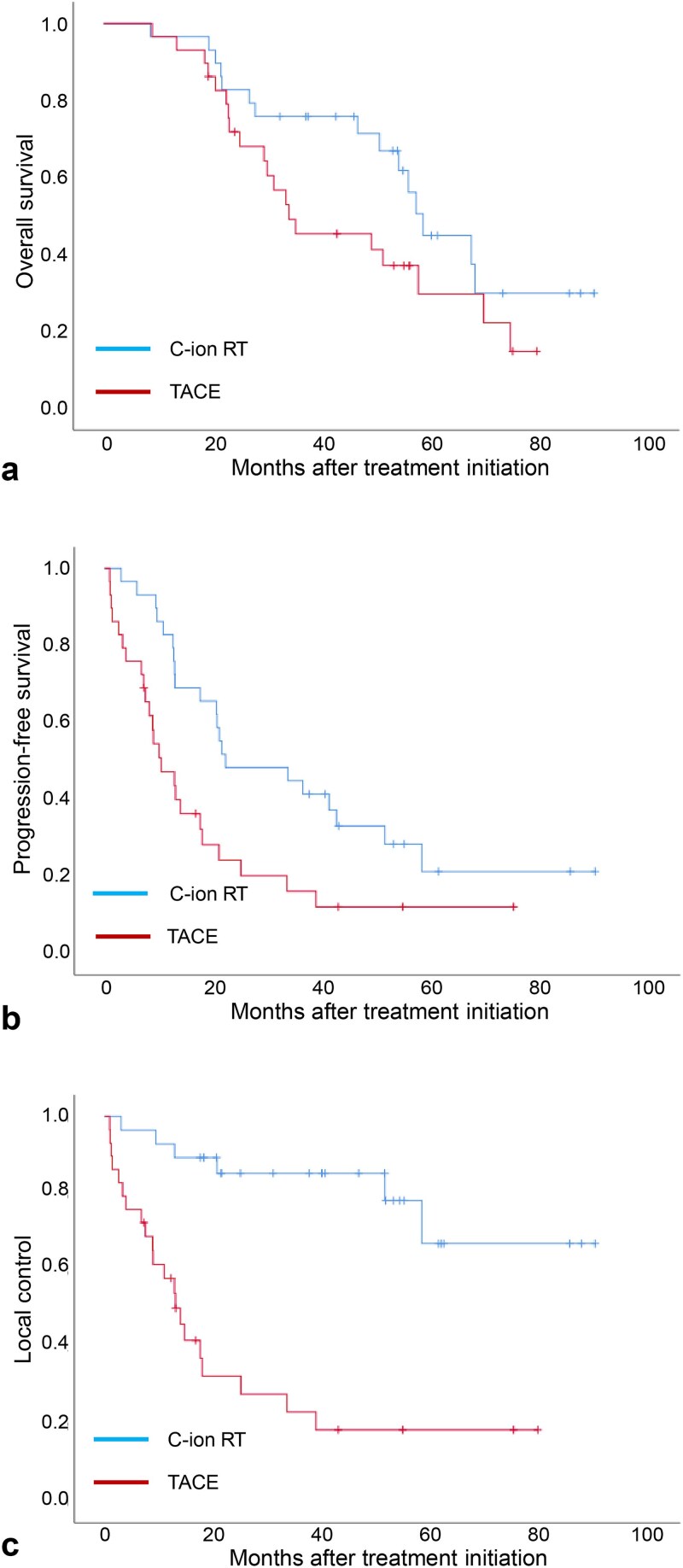
Kaplan–Meier estimates of (a) overall survival, (b) progression-free survival and (c) local control in the matched cohort. C-ion RT = carbon ion radiotherapy, TACE = transarterial chemoembolization.

The Kaplan–Meier curves for OS, PFS and LC in the matched cohort are shown in Fig. 2. The median OS in the C-ion RT group was longer than that in the TACE group (58.6 vs 34.0 months, respectively). The 1- and 3-year OS rates were 96.6% and 75.9% in the C-ion RT group, and 96.6% and 45.4% in the TACE group (HR, 0.578 [95% CI: 0.295–1.132]; *P* = 0.106), respectively. The median PFS in the C-ion RT group was longer than that in the TACE group (22.1 vs 10.3 months, respectively). The 1- and 3-year PFS rates were 82.1% and 48.3% in the C-ion RT group, and 47.2% and 16.1% in the TACE group (HR, 0.460 [95% CI: 0.254–0.835]; *P* = 0.009), respectively. The median time to LC in the C-ion RT group was longer than that in the TACE group (not reached vs 13.9 months, respectively). The 1- and 3-year LC rates were 92.6% and 85.2% in the C-ion RT group, and 57.9% and 23.2% in the TACE group (HR, 0.155 [95% CI: 0.062–0.390]; *P* < 0.001), respectively.

### Prognostic factors

Prognostic factors for OS in the entire cohort are shown in Table 2. Univariate analysis showed that male sex, tumor size <6 cm and C-ion RT were associated with longer OS. In multivariate analysis, male sex, Child–Pugh class A and tumor size <6 cm were independently associated with longer OS.

**Table 2:** Univariate and multivariate analysis of factors associated with overall survival

	Univariate analysis		Multivariate analysis	
	HR (95% CI)	*P*-value	HR (95% CI)	*P*-value
Age (years) (<75 vs ≥75)	0.763 (0.417–1.397)	0.381	0.723 (0.385–1.358)	0.313
Sex (male vs female)	0.478 (0.246–0.930)	**0.030**	0.414 (0.207–0.828)	**0.013**
Etiology (viral vs non-viral)	0.805 (0.459–1.411)	0.449	0.734 (0.379–1.422)	0.359
Child–Pugh class (A vs B)	0.478 (0.212–1.078)	0.075	0.371 (0.153–0.900)	**0.028**
Tumor size (cm) (<6 vs ≥6)	0.408 (0.234–0.712)	**0.002**	0.377 (0.209–0.678)	**0.001**
AFP (ng/mL) (<20 vs ≥20)	0.601 (0.332–1.087)	0.092	0.794 (0.411–1.536)	0.494
Treatment modality (C-ion RT vs TACE)	0.470 (0.271–0.816)	**0.006**	0.559 (0.305–1.026)	0.061

Bold values indicate statistical significance (*P* < 0.05). AFP = alpha-fetoprotein, C-ion RT = carbon ion radiotherapy, TACE = transarterial chemoembolization.

Prognostic factors for PFS in the entire cohort are shown in Table 3. Univariate analysis showed that tumor size <6 cm and C-ion RT were associated with longer PFS. In multivariate analysis, tumor size <6 cm, alpha-fetoprotein (AFP) <20 ng/mL and C-ion RT were independently associated with longer PFS.

**Table 3:** Univariate and multivariate analysis of factors associated with progression-free survival

	Univariate analysis		Multivariate analysis	
	HR (95% CI)	*P*-value	HR (95% CI)	*P*-value
Age (years) (<75 vs ≥75)	1.068 (0.665–1.714)	0.786	1.050 (0.642–1.718)	0.845
Sex (male vs female)	0.987 (0.541–1.800)	0.965	1.116 (0.589–2.114)	0.736
Etiology (viral vs non-viral)	0.895 (0.558–1.437)	0.647	0.910 (0.543–1.525)	0.720
Child–Pugh class (A vs B)	0.738 (0.352–1.544)	0.420	1.000 (0.457–2.185)	0.999
Tumor size (cm) (<6 vs ≥6)	0.514 (0.320–0.824)	**0.006**	0.486 (0.298–0.793)	**0.004**
AFP (ng/mL) (<20 vs ≥20)	0.622 (0.380–1.018)	0.059	0.543 (0.313–0.941)	**0.030**
Treatment modality (C-ion RT vs TACE)	0.549 (0.342–0.882)	**0.013**	0.562 (0.341–0.926)	**0.024**

Bold values indicate statistical significance (*P* < 0.05). AFP = alpha-fetoprotein, C-ion RT = carbon ion radiotherapy, TACE = transarterial chemoembolization.

Prognostic factors for LC in the entire cohort are shown in Table 4. Univariate and multivariate analyses showed that tumor size <6 cm, AFP <20 ng/mL and C-ion RT were associated with better LC.

**Table 4:** Univariate and multivariate analysis of factors associated with local control

	Univariate analysis		Multivariate analysis	
	HR (95% CI)	*P*-value	HR (95% CI)	*P*-value
Age (years) (<75 vs ≥75)	1.072 (0.584–1.969)	0.822	1.210 (0.642–2.281)	0.556
Sex (male vs female)	0.664 (0.327–1.348)	0.258	0.962 (0.434–2.134)	0.962
Etiology (viral vs non-viral)	1.688 (0.940–3.033)	0.080	1.214 (0.631–2.333)	0.561
Child–Pugh class (A vs B)	1.122 (0.400–3.146)	0.827	1.707 (0.580–5.019)	0.331
Tumor size (cm) (<6 vs ≥6)	0.461 (0.254–0.838)	**0.011**	0.445 (0.237–0.834)	**0.012**
AFP (ng/mL) (<20 vs ≥20)	0.445 (0.242–0.820)	**0.009**	0.434 (0.212–0.889)	**0.023**
Treatment modality (C-ion RT vs TACE)	0.260 (0.144–0.469)	**<0.001**	0.282 (0.150–0.528)	**<0.001**

Bold values indicate statistical significance (*P* < 0.05). AFP = alpha-fetoprotein, C-ion RT = carbon ion radiotherapy, TACE = transarterial chemoembolization.

### Dose-dependent effects of carbon ion radiotherapy on local control

The median time to LC was longer in the 4-fraction group than in the 12-fraction group (not reached vs 51.4 months, respectively). The 1- and 3-year LC rates were 92.2% and 68.7% in the 4-fraction group, and 88.9% and 51.6% in the 12-fraction group (HR, 1.715 [95% CI: 0.661–4.449]; *P* = 0.262), respectively.

### Liver function

**Fig. 3:**
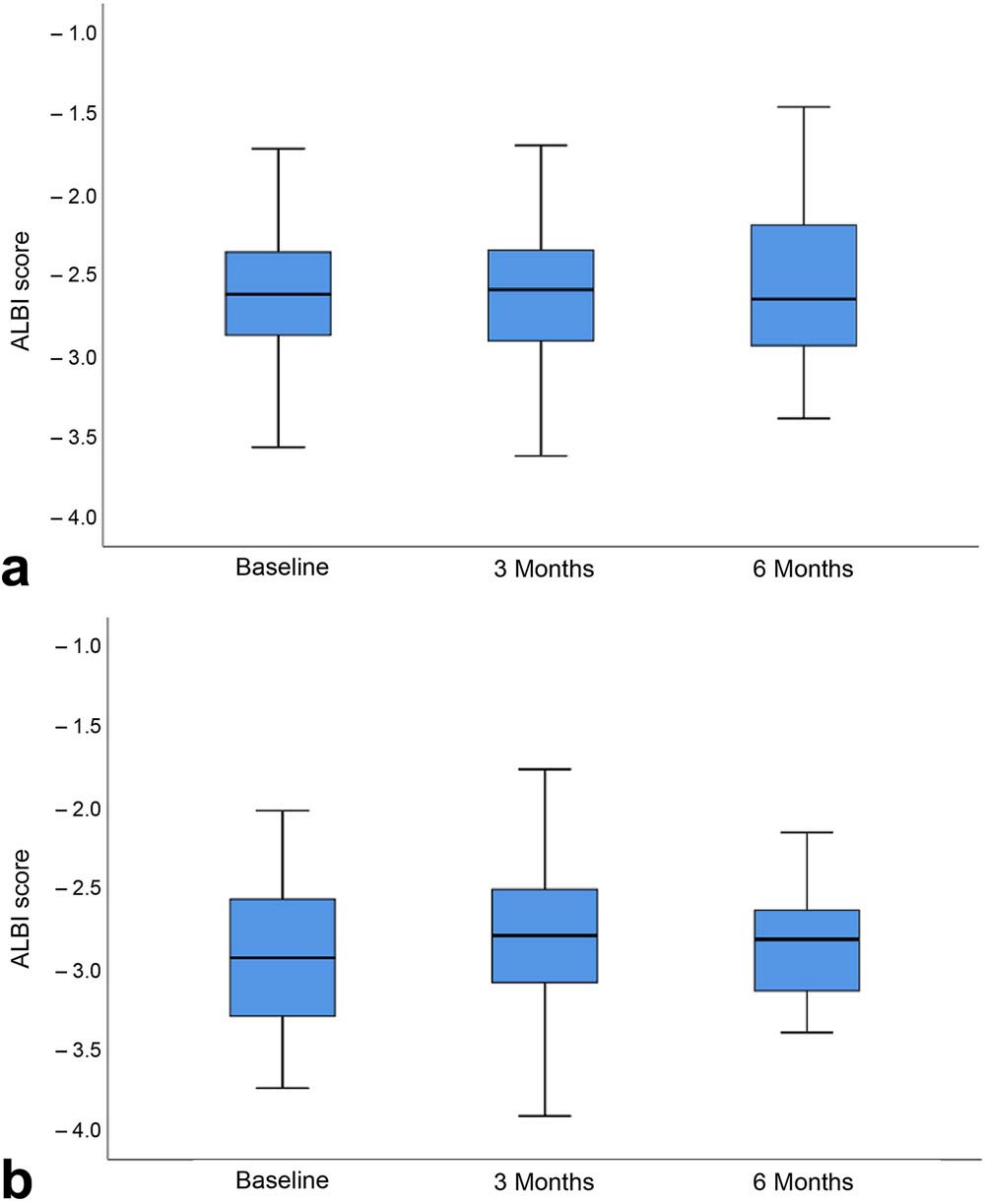
Changes in albumin–bilirubin scores during treatment with (a) carbon ion radiotherapy and (b) transarterial chemoembolization.

Changes in albumin–bilirubin scores following treatment for both groups in the entire cohort are shown in Fig. 3. In the C-ion RT group, albumin–bilirubin scores before, after 3 months and after 6 months were −2.62, −2.59 and −2.65, respectively. No changes were observed after 3 (*P* = 0.959) or 6 months (*P* = 0.887) compared with the start of treatment. In the TACE group, albumin–bilirubin scores before, after 3 months and after 6 months were −2.60, −2.49 and −2.51, respectively. No changes were observed after 3 (*P* = 0.306) or 6 months (*P* = 0.504) compared with the start of treatment. These results suggest that, in both groups, there was no deterioration in albumin–bilirubin scores at 3 and 6 months after treatment. Changes in delta values from baseline were used to assess post-treatment liver function on an individual basis. In the C-ion RT group, the median (IQR) delta values at 3 and 6 months were 0.05 (−0.16 to 0.19) and 0.06 (−0.20 to 0.23), respectively. Similarly, in the TACE group, the median (IQR) delta values at 3 and 6 months were 0.07 (−0.02 to 0.36) and 0.23 (−0.08 to 1.80), respectively.

### Progression patterns

During follow-up, 45 and 28 patients in the C-ion RT and TACE groups, respectively, experienced disease progression. In the C-ion RT group, the progression patterns were as follows: local recurrence, 15 (33.3%) cases; intrahepatic recurrence, 20 (44.4%) cases; extrahepatic metastases, 3 (6.6%) cases; both local and intrahepatic recurrence, 3 (6.6%) cases; both local and extrahepatic metastases, 1 (2.2%) case; and both intrahepatic and extrahepatic metastases, 3 (6.6%) cases. The progression patterns in the TACE group were as follows: local recurrence, 16 (57.1%) cases; intrahepatic recurrence, 2 (7.1%) cases; extrahepatic metastases, 3 (10.7%) cases; and both local and intrahepatic recurrence, 7 (25%) cases.

### Sequential treatment

Sequential treatments after C-ion RT and TACE are shown in Table 5. In the C-ion RT group, 45 patients experienced disease progression; 34 received further treatment after C-ion RT. TACE (13 [22.4%] patients) was the most common second-line treatment after C-ion RT. Seven (12.1%) patients received systemic therapy as second-line treatment; 16 (27.6%) received systemic therapy for all sequential treatments after C-ion RT. In the TACE group, 28 patients experienced disease progression; 24 received further treatment after TACE. Systemic therapy was the most common second-line treatment after TACE. Sixteen (47.1%) patients received systemic therapy as second-line treatment; 17 (50.0%) received systemic therapy for all sequential treatments after TACE. Twenty-six (76.5%) patients received additional TACE for residual lesions. The median interval between the first and second TACE was 87 days.

**Table 5:** Sequential treatment

	Second treatment		All sequential treatments	
	C-ion RT (*n* = 58)	TACE (*n* = 34)	C-ion RT (*n* = 58)	TACE (*n* = 34)
Progression after C-ion RT or TACE, *n* (%)	45 (77.6)	28 (82.4)		
C-ion RT, *n* (%)	5 (8.6)	3 (8.8)	7 (12.1)	3 (8.8)
TACE, *n* (%)	13 (22.4)	0 (0.0)	14 (24.1)	3 (8.8)
Resection, *n* (%)	1 (1.7)	1 (2.9)	2 (3.4)	1 (2.9)
RFA, *n* (%)	7 (12.1)	2 (5.9)	7 (12.1)	2 (5.9)
Radiotherapy, *n* (%)	1 (1.7)	1 (2.9)	1 (1.7)	2 (5.9)
Systemic therapy, *n* (%)	7 (12.1)	16 (47.1)	16 (27.6)	17 (50.0)
HAIC, *n* (%)	0 (0.0)	1 (2.9)	0 (0.0)	2 (5.9)
Best supportive care, *n* (%)	11 (18.9)	4 (11.8)	11 (18.9)	4 (11.8)
No progression after C-ion RT or TACE, *n* (%)	13 (22.4)	6 (17.6)		

C-ion RT = carbon ion radiotherapy, HAIC = hepatic arterial infusion chemotherapy, OS = overall survival, RFA = radiofrequency ablation, TACE = transarterial chemoembolization.

## DISCUSSION

According to current guidelines, the recommended standard of care for solitary HCC is either resection or RFA [4–6]. However, there are some cases where resection is challenging due to considerations of liver function and procedural tolerance. RFA may be a treatment option in such cases. Nevertheless, because the treatment efficacy of RFA tends to decrease with tumor size, it is often avoided for lesions >3 cm [15, 16]. Thus, for solitary HCC >3 cm, if resection is not feasible, TACE is the standard of care. However, as the efficacy of TACE can be suboptimal, and given the need for alternative treatments, we focused on C-ion RT. In this study, we investigated clinical outcomes following C-ion RT and TACE for HCCs >3 cm. We found that the C-ion RT group had better OS, PFS and LC rates than the TACE group for HCCs >3 cm. To our knowledge, no previous study has directly compared C-ion RT and TACE for HCCs >3 cm.

Several studies have been published on C-ion RT [8–14], beginning with the first prospective study by Kato *et al*. [8] in which C-ion RT was reported to have 90.5–97.0% and 50.0–76.7% 1- and 3-year OS rates, respectively; and 92.0–100.0% and 76.5–91.4% 1- and 3-year LC rates, respectively. Notably, our study cohort, with a median age of 78 years and a median tumor size of 49 mm, involved patients of more advanced age and larger sized tumors compared with previous cohorts. In this study, the C-ion RT group demonstrated 3-year OS rates of 78.3% and 3-year LC rates of 64.7%. The LC rates were slightly lower than those reported in previous studies. Shibuya *et al*. [11] reported the outcomes of 4-fraction C-ion RT for HCCs >3 cm, demonstrating favorable results with a 2-year LC rate of 92.3%. However, this study included a higher proportion of patients with lesions adjacent to the gastrointestinal tract and those treated with 12-fraction C-ion RT. In this study, the 4-fraction C-ion RT group demonstrated better LC than the 12-fraction C-ion RT group. Other studies have shown a 2-year LC rate of 81.5% for HCCs >3 cm [13], consistent with our findings. Shiba *et al*. [23] reported a 3-year LC rate of 71.0% for C-ion RT in a comparative study with TACE. However, the Shiba *et al*. [23] study included HCCs <3 cm. The difference in LC rates before and after propensity score matching may have been influenced by the decrease in the percentage of giant HCCs (≥9 cm) after matching. Another contributing factor is the absence of a clearly defined standard for assessing LC after C-ion RT, making evaluation challenging. In this study, local recurrence was independently evaluated by the attending physician and radiation oncologist. Two different evaluators assessed each case, potentially introducing interobserver variability. Assessment is further complicated by persistent tumor enhancement after C-ion RT. This remains a limitation of this study.

Few studies have investigated the clinical outcomes of C-ion RT compared with those of locoregional therapy for HCC. Fujita *et al*. [24] compared the clinical outcomes of C-ion RT and RFA in patients with early-stage HCC. They reported that C-ion RT provided a cumulative local recurrence rate, PFS and OS comparable to those of RFA. They concluded that C-ion RT may be an effective treatment option for early HCC when RFA is contraindicated. Only one study has compared the effectiveness of C-ion RT and TACE. Shiba *et al*. [23] reported that TACE significantly prolonged OS, PFS and LC compared with C-ion RT; however, their cohort included small HCCs with a median tumor diameter of 3 cm, which is a different target from that of our study. For solitary HCC <3 cm, RFA is often selected in real-world clinical settings when resection is not feasible, based on previous studies showing that the outcomes of resection and RFA are comparable [25, 26]. Even for HCCs <3 cm, RFA may not be feasible due to anatomical factors such as proximity to major blood vessels or bile ducts. However, as the decision to perform RFA can vary depending on the operator’s experience, we focused on HCCs >3 cm.

Although multivariate analysis identified C-ion RT as a factor influencing PFS and LC, C-ion RT was not identified as a factor influencing OS. One possible reason for this may be the influence of sequential treatment after recurrence. Multiple treatment options are available after HCC recurrence that have a significant effect on prognosis. In particular, the clinical efficacy of systemic chemotherapy has improved in recent years, with seven different chemotherapy options available in Japan, including sorafenib, regorafenib, lenvatinib, ramucirumab, cabozantinib, atezolizumab plus bevacizumab and tremelimumab plus durvalumab. Several studies have reported the beneficial effects of systemic chemotherapy in TACE-refractory HCC [27–29]. Thus, one possible explanation is that sequential therapy after TACE prolonged post-progression survival in the TACE group.

In this study, the 1- and 3-year OS, PFS and LC rates were 94.1% and 44.6%, 49.2% and 14.5%, and 58.3% and 20.4%, respectively, in the TACE group. Our findings are consistent with previous findings when considering patient demographics and tumor characteristics. Sapir *et al*. [30] reported 1- and 2-year OS and LC rates of 75.3% and 54.9% and 47.1% and 22.9%, respectively, in patients with predominantly solitary HCC. Lee *et al*. [31] reported a 3-year OS rate of 72.8%, and a median time to progression of 8.9 months, in patients with a large solitary HCC (≥5 cm). Similarly, Kim *et al*. [20] demonstrated a median OS and PFS of 48 and 22 months, respectively, in patients with solitary HCC ≥5 cm. The median age of our cohort was 79 years, significantly older than those (61.5–63.0 years) reported in the aforementioned studies. Advanced age may be associated with limited TACE intensity and fewer subsequent treatments, potentially influencing outcomes.

Interestingly, in our study, only 27.6% of patients in the C-ion RT group received systemic chemotherapy as sequential treatment compared with 50.0% of patients who received TACE. These differences in the rates of systemic chemotherapy initiation can be attributed to two primary factors. First, there were differences in the treatment options available after relapse. C-Ion RT, suitable for solitary lesions, is challenging when multiple recurrent lesions occur. Systemic chemotherapy is often administered in cases of tumor recurrence after TACE. Conversely, because TACE is suitable for multiple recurrent lesions, it is often preferred over systemic chemotherapy in cases of recurrence after C-ion RT. Second, at the data cutoff point of this study, the TACE group had a higher recurrence rate. With a prolonged observation period, if recurrence in the C-ion RT group increases, the rate of systemic chemotherapy may also increase. It is also necessary to consider the differences in progression patterns; however, there was no significant difference in the proportion of extrahepatic metastases primarily targeted for systemic chemotherapy between the two groups. Considering the low rate of systemic chemotherapy initiation in the C-ion RT group, this study’s findings indicate that C-ion RT has the potential to extend prognosis without systemic chemotherapy.

No apparent deterioration in liver function was observed at 3 and 6 months after C-ion RT. While previous studies have shown no long-term deterioration in liver function after C-ion RT [10, 12, 14], our study differed in that we focused on HCCs >3 cm. We also examined dose–volume histogram parameters. The mean liver dose was 12.67 Gy (median, 11.29 Gy [range, 4.50–29.85 Gy]), and the V_20Gy_ was 22.69% (median, 19.81% [range, 7.57–56.73%]). Compared to previous studies such as the Abe *et al*. [32] study, which reported a mean liver dose of 8.1 ± 1.4 Gy for C-ion RT, our study showed a higher mean liver dose and V_20Gy_, likely due to larger tumors and different fractionation schemes (12 vs 4). Despite higher doses, no significant changes in liver function were observed based on albumin–bilirubin scores. The results of this study are important from a clinical point of view because liver function does not deteriorate even when the irradiation field is increased. As HCC commonly requires repeated treatment, it is imperative to choose a curative treatment strategy that minimizes adverse effects on liver function.

Stereotactic body radiation therapy for the locoregional treatment of small solitary HCC has been reported to result in favorable treatment outcomes. It serves as a crucial alternative, similar to C-ion RT, in patients for whom resection or RFA is not feasible. Results from prospective stereotactic body radiation therapy trials have indicated 2- and 3-year OS rates of 69–84% and 66–78%, respectively, and 2- and 3-year LC rates of 90–97% and 90–96%, respectively [33–36]. Recent studies using propensity score matching have compared stereotactic body radiation therapy with RFA and have shown stereotactic body radiation therapy to be significantly associated with a lower risk of local recurrence than RFA [37, 38]. However, these studies primarily focused on small HCCs (median tumor size, 14–30 mm), with limited evidence in relation to medium-to-large-sized HCCs. C-Ion RT has the distinctive property of a steep dose distribution, suggesting a minimal effect on hepatic function, even in large HCCs. In contrast, stereotactic body radiation therapy for large tumors is often challenging because of potential damage to normal liver tissues [32]. Consequently, while a direct comparison between stereotactic body radiation therapy and C-ion RT has not been conducted, C-ion RT may yield higher treatment efficacy, especially for medium-to-large-sized HCCs. Proton beam radiotherapy could also be an alternative to TACE. Bush *et al*. [39] conducted a randomized trial comparing the efficacy of proton beam radiotherapy and TACE. They reported that while OS was comparable between the two groups, PFS and LC were significantly better in the proton beam radiotherapy group than in the TACE group. Bush *et al*. [39] showed that patients treated with proton beam radiotherapy required fewer treatment sessions, experienced shorter post-treatment hospitalization and incurred lower overall treatment costs compared to those treated with TACE. Proton beam radiotherapy and C-ion RT share similar properties due to the Bragg peak phenomenon.

A major limitation of this study was that it was a retrospective observational study involving patients from a single center with limited sample sizes. The decision to perform C-ion RT or TACE depends on several factors, such as age, tumor size, liver function and concomitant diseases. We attempted to reduce this bias by using propensity score matching. However, the matching was not perfect, and unavoidable bias may have persisted. In particular, the limited number of cases made it difficult to perform sufficient statistical analyses such as multivariate analysis and propensity score matching. Statistical analyses with small sample sizes require both caution when interpreting our results and verification in larger studies. Another limitation is the considerable disparity in medical costs between C-ion RT and TACE in Japan. While C-ion RT became eligible for insurance coverage in Japan for solitary HCC >4 cm in 2022, our study period predated this policy change. Consequently, C-ion RT incurred substantial costs for patients; hence, the inclusion of only patients in the C-ion RT group who could bear the high medical costs suggests the potential for socioeconomic bias. Socioeconomic bias may have a greater impact on OS than PFS or LC; however, no difference in OS was observed. A wide range of treatment options are available for HCC, including locoregional and systemic therapies. Most treatments are covered by insurance in Japan. Therefore, the ability to proceed to subsequent treatment at a lower cost may be one reason for the lack of differences in OS.

In conclusion, C-ion RT provided better OS, PFS and LC than TACE in patients with solitary HCC >3 cm. Our results emphasize the need for a prospective randomized trial comparing the efficacy of C-ion RT with that of TACE. This study indicated that C-ion RT is a possible alternative to TACE, which is the standard of care for patients with medium-to-large-sized HCCs.

## Data Availability

The data that support the findings of this study are available on request from the corresponding author, T.F. The data are not publicly available due to privacy or ethical restrictions.
